# Sevoflurane Preconditioning plus Postconditioning Decreases Inflammatory Response with Hemodynamic Recovery in Experimental Liver Ischemia Reperfusion

**DOI:** 10.1155/2019/5758984

**Published:** 2019-04-07

**Authors:** Estela Regina Ramos Figueira, Joel Avancini Rocha-Filho, Cinthia Lanchotte, Ana Maria Mendonça Coelho, Mauro Nakatani, Eduardo Ryoiti Tatebe, Jonathan Augusto Venceslau Lima, Camilla Oliveira Mendes, Bruno Camargo Rocha Paim de Araujo, Emilio Elias Abdo, Luiz Carneiro D'Albuquerque, Flavio Henrique Ferreira Galvão

**Affiliations:** ^1^Department of Gastroenterology, Division of Digestive Surgery, Hospital das Clinicas of University of Sao Paulo School of Medicine, Sao Paulo, Brazil; ^2^Department of Gastroenterology, Medical Investigation Laboratory LIM37, Hospital das Clinicas of University of Sao Paulo School of Medicine, Sao Paulo, Brazil; ^3^Discipline of Anesthesiology, Hospital das Clinicas of University of Sao Paulo School of Medicine, Sao Paulo, Brazil; ^4^Medical Student and Scientific Research Initiation in Medicine at University of Sao Paulo School of Medicine, Brazil; ^5^Department of Gastroenterology, Division of Liver and Gastrointestinal Transplantation, Hospital das Clinicas of University of Sao Paulo School of Medicine, Sao Paulo, Brazil

## Abstract

**Objective:**

The inhalation anesthetic sevoflurane has presented numerous biological activities, including anti-inflammatory properties and protective effects against tissue ischemic injury. This study investigated the metabolic, hemodynamic, and inflammatory effects of sevoflurane pre- and postconditioning for short periods in the rescue of liver ischemia-reperfusion (IR) injury using a rat model.

**Materials and Methods:**

Twenty Wistar rats were divided into four groups: sham group, control ischemia group (partial warm liver ischemia for 45 min followed by 4 h of reperfusion), SPC group (administration of sevoflurane 2.5% for 15 min with 5 min of washout before liver IR), and SPPoC group (administration of sevoflurane 2.5% for 15 min before ischemia and 20 min during reperfusion).

**Results:**

All animals showed a decrease in the mean arterial pressure (MAP) and portal vein blood flow during ischemia. After 4 h of reperfusion, only the SPPoC group had MAP recovery. In both the SPC and SPPoC groups, there was a decrease in the ALT level and an increase in the bicarbonate and potassium serum levels. Only the SPPoC group showed an increase in the arterial blood ionized calcium level and a decrease in the IL-6 level after liver reperfusion. Therefore, this study demonstrated that sevoflurane preconditioning reduces hepatocellular injury and acid-base imbalance in liver ischemia. Furthermore, sevoflurane postconditioning promoted systemic hemodynamic recovery with a decrease in inflammatory response.

## 1. Introduction

Ischemia-reperfusion (IR) injury is still a major problem that impairs liver transplantation (LT) and surgery outcome. Multiple inflammatory and metabolic pathways that are involved in liver IR injury are still unclear [1]. Currently, strategies applied to decrease liver IR injury have obtained only partial response with scant application in the clinical setting due to side effects. In this setting, volatile anesthetics (VAs) such as sevoflurane, which are commonly used in clinical practice, arise as a promising strategy to prevent IR injury [2, 3].

Some studies on VA conditioning in liver IR show conflicting results in the reduction of liver injury [4]. Jeong et al. [5] demonstrated a decrease in the AST and ALT levels after 45 min of liver ischemia using either sevoflurane or isoflurane anesthesia in association with ischemic preconditioning (IPC) for 10 min. In contrast, in liver surgery, pharmacological postconditioning with sevoflurane decreases liver injury with better patient outcome comparable with the results of intermittent clamping [6], and similar benefits are observed with preconditioning [7]. In LT, Minou et al. [8] showed that sevoflurane preconditioning applied during deceased donor surgery decreases postoperative AST peak and incidence of early graft dysfunction.

Presently, sevoflurane and other VAs have been widely used in general anesthesia. However, the beneficial effects of sevoflurane conditioning in liver IR still need additional data. It is possible that a shorter sevoflurane application, during short periods, is still beneficial in decreasing liver IR injury. Some authors have suggested that the protective effects of VAs occur through mechanisms similar to IPC protection [3, 6]. Adamczyk et al. [9], using a model of brain ischemia, suggested that sevoflurane protection may be mediated through mitochondrial potassium (K) ATP-dependent channel opening analogous to the IPC mechanism. Previously, we demonstrated the positive effects of IPC on the restoration of the portal vein blood flow and the metabolic profile after liver IR [10], raising a question concerning sevoflurane effects on hemodynamic profile during liver IR. In this study, we investigate the metabolic, hemodynamic, and inflammatory effects of sevoflurane pre- and postconditioning for short periods in the rescue of liver IR injury using a rat model.

## 2. Methods

### 2.1. Animals

Twenty male Wistar rats weighing 280–300 g were housed in individual cages at 12 h light/dark cycle under a temperature of 20–24°C, humidity of 50–60%, and ultrafiltered air, receiving rat chow and water ad libitum. The study was conducted with approval from the Ethics Committee on Animal Use (CEUA) of the University of Sao Paulo School of Medicine (no. 238/11). Animals received care in accordance with the Guide for the Care and Use of Laboratory Animals [11].

### 2.2. Experimental Design

**Figure 1:**
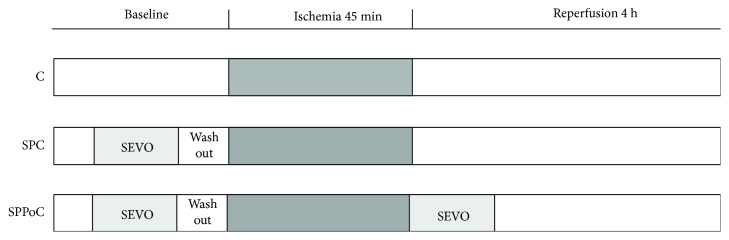
Study groups: C (control) group, animals subjected to liver ischemia; SPC (sevoflurane preconditioning) group, animals treated with 2.5% sevoflurane for 15 min followed by 5 min of washout before ischemia; and SPPoC (sevoflurane pre- plus postconditioning) group, animals treated with sevoflurane for 15 min before ischemia and for additional 20 min following reperfusion.

Rats were randomly allocated into four groups according to Figure 1. In the sham group (*n* = 5), we performed midline laparotomy and liver manipulation without IR; in the control (C) ischemia group (*n* = 5), animals that did not receive VA (ketamine 100 mg/kg and xylazine 10 mg/kg) were subjected to liver ischemia for 45 min; in the sevoflurane preconditioning (SPC) group (*n* = 5), animals received sevoflurane (Abbott Laboratories, USA) for 15 min followed by 5 min of washout before liver ischemia; and in the sevoflurane pre- and postconditioning (SPPoC) group (*n* = 5), animals received sevoflurane before liver ischemia as in the SPC group and 20 min of sevoflurane after reperfusion. Sevoflurane was administered using a calibrated vaporizer (Sevovapor Model 1225, Takaoka, Brazil) at a concentration of 1 MAC with an expiratory fraction of 2.5%.

### 2.3. Surgery Procedure and Sample Collection

Animals received intraperitoneal anesthesia through an injection of ketamine hydrochloride (Ketalar®, Cristália, Brazil) 30 mg/kg and xylazine (Rompun®, Bayer, Germany) 30 mg/kg. After orotracheal intubation, the rats underwent mechanical ventilation (Small Animal Ventilator model 6839 from Harvard Apparatus, USA) using a tidal volume of 8 mL air per kg of body weight, with 60 bpm and FiO2 of 21%. A rectal digital thermometer (YSI Precision 4000A, USA) was used to monitor the body temperature maintained at 35–37°C until completion of the procedure. The right common carotid artery was catheterized with PE50 for hemodynamic measurements and blood sampling.

After performing midline laparotomy, the common pedicle of the median and the left lateral hepatic lobes was occluded with an atraumatic microclamp. Partial hepatic ischemia involving the median and left lateral lobes with preservation of right and caudate lobes produces ischemia of 70–80% of the liver without provoking splanchnic congestion. After 45 min of ischemia, the microclamp was removed allowing hepatic reperfusion, which was followed by an immediate removal of nonischemic lobes [10, 12]. To collect hemodynamic data, the animals were maintained anesthetized under mechanical ventilation throughout the reperfusion period, being hydrated via subcutaneous injection of NaCl 0.9%, 10 mL/kg/h. After completing 4 h of reperfusion, the arterial blood (via the carotid artery) and the liver samples were collected. Animals were euthanized through exsanguination.

### 2.4. Hemodynamics and Portal Blood Flow

The MP150 Starter System (BIOPAC Systems Inc., USA) registered the systolic and diastolic blood pressures and the mean arterial pressure (MAP, mmHg) via the carotid artery. A perivascular probe (Transonic Systems Inc., USA) connected to a flowmeter (TS420 Animal Research, Transonic Systems Inc., USA) measured the portal vein blood flow. These parameters were recorded from the three ischemic groups at five periods: after induction of anesthesia (baseline), 5 min after induction of liver ischemia (I5), immediately before reperfusion (PR), 5 min after starting reperfusion (POR5), and 4 h after starting reperfusion (POR4h).

### 2.5. Analysis of Transaminases

The optimized ultraviolet method (Cobas Mira, Roche Diagnostics, Switzerland) was used to determine the AST and ALT levels.

### 2.6. Biochemical Analysis

Bicarbonate (BIC), base excess (BE), pH, lactate, ionized calcium (iCa), K, and glucose levels were analyzed in the arterial blood samples through the ABL800 FLEX blood gas analyzer (Radiometer Medical ApS, Denmark).

### 2.7. Analysis of Cytokines

Commercially available enzyme-linked immunosorbent assay (ELISA) kits from Invitrogen (Thermo Fisher Scientific, USA) were used to determine the TNF-*α*, IL-6, and IL-10 serum levels.

### 2.8. Analysis of Lipid Peroxidation

Malondialdehyde (MDA) content was analyzed as a parameter for lipid peroxidation as described previously [13]. Briefly, 100 mg/mL of ischemic liver samples was homogenized in 1.15% KCl buffer and centrifuged for 20 min at 14,000 × *g*. The supernatant was added to a reaction mixture of 1.5 mL thiobarbituric acid, sodium dodecyl sulfate, acetic acid (pH 3.5), and distilled water. The mixture was heated at 90°C for 45 min and cooled at room temperature. Then, the samples were submitted to centrifugation (10,000×*g* for 10 min), and absorbance at 532 nm was measured. Lipid peroxide content is expressed as nmol of MDA/mg of protein.

### 2.9. Statistical Analysis

Results expressed as mean ± SD were analyzed using unpaired Student's *t*-test. A *p* value of <0.05 was considered significant. Statistical analysis was performed using the Prism 6 software (GraphPad, USA).

## 3. Results

### 3.1. Hemodynamics and Portal Blood Flow

**Figure 2:**
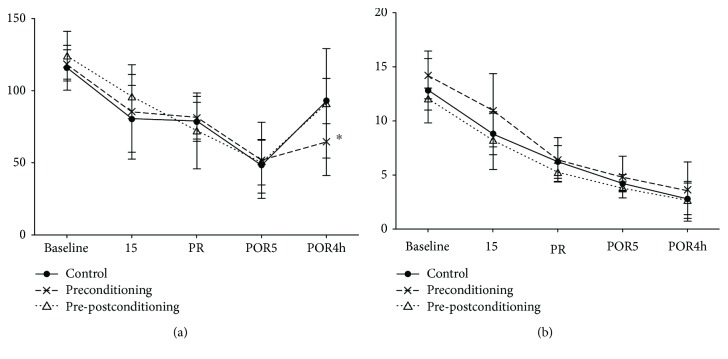
Hemodynamics: (a) mean arterial pressure (MAP, mmHg) and (b) portal blood flow (PBF, mL/min) at baseline; I5, 5 min after induction of liver ischemia; PR, immediately before reperfusion; POR5, 5 min after start of reperfusion; and POR4h, 4 hours after start of reperfusion. All 3 groups show that baseline MAP (a) is increased compared to I5, PR, POR5, and POR4h (*p* < 0.05), except for baseline compared to POR4h MAP in the control group. At 4 h after reperfusion (POR4h), MAP is increased in the pre- plus postconditioning group compared to the preconditioning group (^∗^*p* < 0.05). All 3 groups show that baseline PBF (b) is increased compared to I5, PR, POR5, and POR4h (*p* < 0.05), except for baseline compared to I5 PBF in the preconditioning group. Values are represented as means with SD.

MAP decreased in all groups during ischemia until 5 min of reperfusion. At 4 h after reperfusion, MAP recovered in the C group (91.4 ± 37.96 vs. 115.6 ± 15.57 mmHg) and decreased in the SPC (64.6 ± 23.38 vs. 118.2 ± 37.06 mmHg) and SPPoC (93.0 ± 15.73 vs. 124.0 ± 17.28 mmHg) groups compared to the baseline. However, MAP increased in the SPPoC group compared to that in the SPC group (93 ± 15.73 vs. 64.6 ± 23.38 mmHg, Figure 2(a)), indicating some recovery of blood pressure. Portal vein blood flow decreased significantly during ischemia and continued to decrease 4 h after reperfusion in the C (2.8 ± 1.48 vs. 12.8 ± 2.95 mL/min), SPC (3.6 ± 2.61 vs. 14.2 ± 2.28 mL/min), and SPPoC (2.6 ± 1.82 vs. 12.0 ± 1.0 mL/min) groups compared to that at the baseline (Figure 2(b)).

### 3.2. Analysis of Transaminases

**Figure 3:**
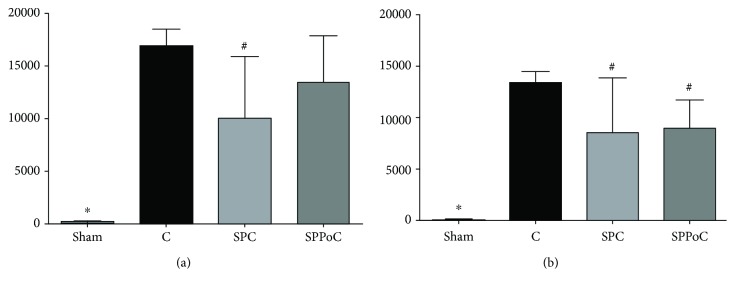
Liver transaminases at 4 hours after reperfusion: (a) AST: aspartate transaminase and (b) ALT: alanine transaminase. Groups: C (control) group; SPC (sevoflurane preconditioning) group; SPPoC (sevoflurane pre- plus postconditioning) group. ^∗^*p* < 0.05 compared to any other group; ^#^*p* < 0.05 compared to the C group.

At 4 h of reperfusion, the AST and ALT levels increased significantly in all the three groups submitted to liver IR compared to those in the sham group. The AST level (Figure 3(a)) decreased in the SPC group (10,056 ± 5,830 U/L, *p* = 0.0178) compared to that in the C group (16,890 ± 1,630 U/L). The ALT level (Figure 3(b)) decreased in the SPC (8,586 ± 5,296 U/L, *p* = 0.0404) and SPPoC (8,956 ± 2,790 U/L, *p* = 0.0052) groups compared to that in the C group (13,418 ± 1,088 U/L).

### 3.3. Biochemical Analysis

Levels of pH, BIC, BE, and glucose in the arterial blood decreased and lactate and K levels increased in all the groups compared to those in the sham group at 4 h after reperfusion, showing deterioration of metabolic profile. However, BIC level increased in both sevoflurane groups, SPC (12.4 ± 4.4 mEq/L, *p* = 0.0242) and SPPoC (11.2 ± 4.31 mEq/L, *p* = 0.0495), compared to that in the C group (6.7 ± 3.3 mEq/L). BE level increased in the SPC group (−14.7 ± 4.48 mEq/L) compared to that in the C group (−20.4 ± 4.22 mEq/L, *p* = 0.0345). The SPPoC (4.81 ± 0.23 mg/dL) group presented an increased iCa level compared to the SPC (4.52 ± 0.21 mg/dL, *p* = 0.034) and C (4.31 ± 0.42 mg/dL, *p* = 0.0241) groups. Serum K level increased in the sevoflurane groups, SPC (6.3 ± 0.95 mEq/L, *p* = 0.0083) and SPPoC (6.12 ± 1.27 mEq/L, *p* = 0.0309), compared to that in the C group (4.7 ± 0.68 mEq/L) (Table 1).

### 3.4. Analysis of Inflammatory Mediators

At 4 h of reperfusion, serum TNF-*α*, IL-6, and IL-10 levels increased significantly in all the three groups submitted to liver IR compared to those in the sham group. However, IL-6 level significantly decreased in the SPPoC group (5,548 ± 2,118 ng/mL) compared to that in the C (7,217 ± 921 ng/mL) and SPC (6,958 ± 746 ng/mL) groups (Table 2).

### 3.5. Lipid Peroxidation Analysis

At 4 h of reperfusion, all groups submitted to liver IR showed significantly increased MDA levels compared to the sham group. MDA content in the sevoflurane groups had no difference with that in the C group (Table 2).

## 4. Discussion

This study evaluated the influence of sevoflurane pre- and postconditioning on hemodynamic behavior during liver IR and the metabolic and inflammatory profiles at 4 h after liver reperfusion. Animals submitted to partial warm liver IR were treated with sevoflurane inhalation either for a few minutes before liver ischemia or before ischemia and during the start of liver reperfusion. Rats treated with either sevoflurane preconditioning alone or pre- and postconditioning administration presented decreased transaminases and increased BIC and K levels, without significant changes in the portal vein flow. Moreover, rats receiving further sevoflurane postconditioning showed better hemodynamic recovery with increased systemic blood pressure, increased serum iCa level, and decreased inflammatory profile, showing lower IL-6 levels.

In this experimental model, the application of partial liver ischemia provided conservation of the portal blood flow. Thus, it prevented the development of splanchnic congestion with its harmful effects on the outcome of animals, which could change the study results as postulated elsewhere [14]. Furthermore, as detailed in our previous study [10], during reperfusion, resection of the right and caudate liver lobes, not subjected to ischemia, reinforces the actual effects of IR injury, abolishing the influence on these healthy lobes, corresponding to 25% to 30% of liver mass, on animal recovery. To exemplify, our previous study on liver IR showed an AST level of >3,000 IU/L without any mortality after 1 h of partial liver ischemia [14].

VAs, mainly isoflurane and sevoflurane, have been broadly used in general anesthesia. However, there is a growing interest in understanding the nonanesthetic effects of these drugs with respect to their proprieties in preventing ischemic organ injury in some clinical situations, such as cardiac infarction, hepatic surgery, and organ transplantation [2, 7, 15]. Freedman et al. [16] showed that enflurane increased postischemic peak ventricular pressure recovery with increased adenosine triphosphate levels, and later in 1989, Kashimoto et al. [17] showed similar effects in an experiment with rat hearts perfused with sevoflurane.

In this study, the use of sevoflurane during short periods, before and after liver ischemia, allowed us to determine if a more restricted application of this drug can still provide beneficial effects on liver IR injury. In sectoring for the first time sevoflurane administration in periods related to the development of hepatic IR, this study attempted to determine the efficacy as a protector after drug application for short periods. Animals showed decreased AST and ALT levels with liver preconditioning with sevoflurane for 15 min. Previously, in this laboratory, Cavalcante et al. [18] showed decreased transaminase levels with continuous sevoflurane anesthesia for about 4 h of the whole experiment. In a randomized study in liver surgery, Beck-Schimmer et al. [7] showed decreased AST and ALT levels in patients preconditioned with sevoflurane before liver ischemia. In LT, Minou et al. [8] showed a significantly decreased AST level and decreased incidence of early allograft dysfunction when liver allografts were preconditioned with sevoflurane during organ donor retrieval. These findings are congruent with those shown in the present study. In contrast, Beck-Schimmer et al. [2] failed to demonstrate a decrease in AST level with sevoflurane postconditioning alone in a study enrolling 98 LT patients, whereas the sevoflurane group presented decreased incidence of severe postoperative complications.

Although some authors questioned the results of VAs when compared to anesthetics such as propofol [19, 20], currently, it has been recognized that VAs have potential effects in decreasing IR injury. Experimental studies showed strong evidence that conditioning with sevoflurane and other VAs significantly reduced IR injury of various organs [3, 21, 22]. Although the complete mechanisms involved in these protective effects remain unclear, some authors have suggested antioxidant and antiapoptotic properties of sevoflurane preconditioning after liver and cerebral IR injuries [23, 24]. Zhang et al. [24] suggested that this antiapoptotic effect is associated with the PI3K/Akt pathway, while the antioxidant activity is mediated via reactive oxygen species signal pathway. They also demonstrated a dose-dependent decrease in TNF-*α*, IL-6, and IL-10 levels. However, Cavalcante et al. [18] failed to demonstrate a decrease in the cytokine levels after conditioning with sevoflurane for 4 h during the experimental liver IR. In the present study, only sevoflurane preconditioning associated to postconditioning induced lower plasma IL-6 levels, without significant changes in IL-10 and TNF-*α* levels. It is possible that the high severity of liver injury, demonstrated by extremely high transaminase levels achieved in the postreperfusion period, masked in some degree the anti-inflammatory activity of sevoflurane.

Some authors suggest that the mechanisms of sevoflurane conditioning involved in decreasing hepatic IR injury are similar to those of IPC [5]. In a study on the heart, Kersten et al. [15] suggested in 1997 that the mechanism of isoflurane protection involves opening of mitochondrial K_atp_ channels similar to that of IPC. More recently, Jiang et al. [25] suggested that the same mechanism is related to sevoflurane postconditioning protection against heart ischemia. Sevoflurane administration and IPC have been related to the decrease in oxidative stress levels following IR injury [23, 26]. However, this study, in accordance with the study by Hsiao et al. [27], failed to demonstrate this effect.

Additionally, in a previous study, we showed that IPC provides better recovery of portal blood flow and metabolic imbalance after liver IR, increasing the iCa and BIC levels [10]. Interestingly, the sevoflurane groups presented a less pronounced acid-base imbalance, showing higher BIC and lower BE levels without significant improvement in the pH level. A low pH level is probably related to the maintenance of higher lactate levels due to the severity of liver injury demonstrated by the critical low glucose levels in all the animal groups, suggesting an impairment of glycogen metabolism [28]. In contrast, sevoflurane increased serum K and iCa levels after 4 h of hepatic reperfusion. Although hyperkalemia in this study may be a side effect of VAs such as sevoflurane (SEVORANE AF Product Monograph–Control no. 209018, AbbVie Corporation, Canada), increased iCa level has been related to attenuation of IR injury [10, 29]. In our previous study, liver IPC induces an increase in serum iCa level 12 h after reperfusion, which is associated with improvement in liver IR injury in connection with other signs of recovery such us decreased liver transaminase and lactate and increased BIC levels [10].

In conclusion, conditioning with sevoflurane provides protection against liver IR, decreasing hepatocellular injury and acid-base imbalance, suggesting a therapeutic option to decrease IR injury in patients undergoing surgical procedures with prolonged interruption of liver inflow. The association of sevoflurane postconditioning with preconditioning increased the protection effect over sevoflurane preconditioning alone, showing an attenuated inflammatory response and increased systemic hemodynamic recovery. Up to now, the intrinsic mechanisms of sevoflurane protection are not entirely clear. In this study, the protective effects of sevoflurane were not related to a decrease in oxidative stress or an increase in portal vein flow.

## Figures and Tables

**Table 1:** Biochemical data at 4 hours after reperfusion.

Variables	Sham	C group	SPC group	SPPoC group
pH	7.4 ± 0.09^∗^	7.17 ± 0.08	7.16 ± 0.10	7.14 ± 0.10
Bicarbonate (mEq/L)	20.6 ± 3.50^∗^	6.7 ± 3.32	12.4 ± 4.39^#^	11.2 ± 4.31^#^
Base excess (mEq/L)	−3.34 ± 4.0^∗^	−20.5 ± 4.22	−14.7 ± 4.48^#^	−16.0 ± 5.19
Lactate (mg/dL)	12 ± 5.26^∗^	66 ± 25.9	43 ± 18.6	74 ± 35.1
Ionized calcium (mg/dL)	4.57 ± 0.43	4.31 ± 0.42	4.52 ± 0.21^&^	4.81 ± 0.23^#^
Potassium (mEq/L)	3.8 ± 0.16^∗^	4.7 ± 0.68	6.3 ± 0.95^#^	6.12 ± 1.27^#^
Glucose (mg/dL)	347 ± 29^∗^	35 ± 18	54 ± 23	51 ± 26

C: control group; SPC: sevoflurane preconditioning group; SPPoC: sevoflurane pre- plus postconditioning group. ^∗^*p* < 0.05 compared to any other group; ^#^*p* < 0.05 compared to the C group; ^&^*p* < 0.05 compared to the SPPoC group.

**Table 2:** Inflammatory mediators at 4 h after reperfusion.

Variables	Sham	C group	SPC group	SPPoC group
MDA (nmol/mg protein)	2.42 ± 1.03^∗^	5.44 ± 1.37	5.52 ± 0.68	5.40 ± 1.06
TNF alpha (pg/mL)	0^∗^	209 ± 72.5	239 ± 131	212 ± 95.5
Interleukin 6 (pg/mL)	218 ± 81.5^∗^	7,217 ± 921^#^	6,958 ± 746^#^	5,548 ± 2,118
Interleukin 10 (pg/mL)	0^∗^	1,555 ± 278	1,481 ± 290	1,332 ± 473

MDA: malondialdehyde; C: control group; SPC: sevoflurane preconditioning group; SPPoC: sevoflurane pre- plus postconditioning group. ^∗^*p* < 0.05 compared to any other group and ^#^*p* < 0.05 compared to SPPoC.

## Data Availability

The data used to support the findings of this study are included within the article.
